# Supplementary material for: The Effects of Exercise during Pregnancy on Gestational Diabetes Mellitus, Preeclampsia, and Spontaneous Abortion among Healthy Women—A Systematic Review and Meta-Analysis
Source: Int J Environ Res Public Health. 2023 Jun 6;20(12):6069. doi: 10.3390/ijerph20126069 (PMC10298745; doi:10.3390/ijerph20126069)
Supplement: Supplementary file 1 [file ijerph-20-06069-s001.zip › ijerph-2324371-supplementary.pdf]

Table S1. Unsorted full-text article.

	Study name	Reason for eksklusion
**1**	Perales M, Refoyo I, Coteron J, Bacchi M, Barakat R. Exercise during pregnancy attenuates prenatal depression: a randomized controlled trial. Eval Health Prof. 2015 Mar;38(1):59-72.	Wrong patient population
**2**	Ramírez-Vélez R, Lobelo F, Aguilar-de Plata AC, Izquierdo M, García-Hermoso A. Exercise during pregnancy on maternal lipids: a secondary analysis of randomized controlled trial. BMC Pregnancy Childbirth. 2017 Nov 28;17(1):396.	Wrong outcomes
**3**	Vargas-Terrones M, Nagpal TS, Perales M, Prapavessis H, Mottola MF, Barakat R. Physical activity and prenatal depression: going beyond statistical significance by assessing the impact of reliable and clinical significant change. Psychol Med. 2021 Mar;51(4):688-693.	Wrong outcomes
**4**	Ctri 2011, The Effects of Yoga in Prevention of Pregnancy Complications in pregnant women who have high chance of developing them	Wrong study design
**5**	Songøygard KM, Stafne SN, Evensen KAI, Salvesen KÅ, Vik T, Mørkved S. Does exercise during pregnancy prevent postnatal depression? A randomized controlled trial. Acta Obstet Gynecol Scand. 2012 Jan;91(1):62-67.	Wrong outcomes
**6**	Okido MM, Valeri FL, Martins WP, Ferreira CH, Duarte G, Cavalli RC. Assessment of foetal wellbeing in pregnant women subjected to pelvic floor muscle training: a controlled randomised study. Int Urogynecol J. 2015 Oct;26(10):1475-81.	Wrong outcomes
**7**	Newham, James & Hurley, Janine & Wittkowski, Anja & Aplin, John & A., Westwood. (2011). Can antenatal yoga lower maternal anxiety (CALMA study): Initial findings.	Wrong outcomes
**8**	Stafne SN, Salvesen KÅ, Romundstad PR, Stuge B, Mørkved S. Does regular exercise during pregnancy influence lumbopelvic pain? A randomized controlled trial. Acta Obstet Gynecol Scand. 2012 May;91(5):552-9.	Wrong outcomes
**9**	Vesco KK, Karanja N, King JC, Gillman MW, Perrin N, McEvoy C, Eckhardt C, Smith KS, Stevens VJ. Healthy Moms, a randomized trial to promote and evaluate weight maintenance among obese pregnant women: study design and rationale. Contemp Clin Trials. 2012 Jul;33(4):777-85.	Wrong outcomes

**10**	Granath AB, Hellgren MS, Gunnarsson RK. Water aerobics reduces sick leave due to low back pain during pregnancy. J Obstet Gynecol Neonatal Nurs. 2006 Jul-Aug;35(4):465-71	Wrong outcomes
**11**	Meher S, Duley L. Exercise or other physical activity for preventing pre-eclampsia and its complications. Cochrane Database Syst Rev. 2006 Apr 19;2006(2):CD005942.	Wrong study design
**12**	Taghiof H, Rezai S, Henderson CE. Effect of an Exercise Intervention on Gestational Diabetes Mellitus: A Randomized Controlled Trial. Obstet Gynecol. 2015 Sep;126(3):676.	Wrong study design (comment to another study)
**12**	Labonte-Lemoyne E, Curnier D, Ellemberg D. Exercise during pregnancy enhances cerebral maturation in the newborn: A randomized controlled trial. J Clin Exp Neuropsychol. 2017 May;39(4):347-354.	Wrong outcomes
**14**	Knowlton, J. R.; Hanson, J.; May, L. Exercise prescription for improved heart rate variability during pregnancy PM and R 2014;6(9 SUPPL. 1):S189 Elsevier Inc. 2014	Wrong study design (poster presentation)
**15**	Dekker Nitert M, Barrett HL, Denny KJ, McIntyre HD, Callaway LK; BAMBINO group. Exercise in pregnancy does not alter gestational weight gain, MCP-1 or leptin in obese women. Aust N Z J Obstet Gynaecol. 2015 Feb;55(1):27-33.	Wrong outcomes
**16**	Haakstad LA, Torset B, Bø K. What is the effect of regular group exercise on maternal psychological outcomes and common pregnancy complaints? An assessor blinded RCT. Midwifery. 2016 Jan;32:81-6.	Wrong outcomes
**17**	Cordero, Yaiza & Pelaez, Mireia & Miguel, Marta & Perales, María & Barakat, Ruben. (2012). ¿Puede el ejercicio físico moderado durante el embarazo actuar como un factor de prevención de la Diabetes Gestacional? (Can moderate physical exercise during pregnancy act as a factor in preventing Gestational Diabetes?). RICYDE. Revista internacional de ciencias del deporte. 8. 3-19. 10.5232/ricyde2012.02701.	languages
**18**	Makhija A, Khatik N, Raghunandan C. A randomized control trial to study the effect of integrated yoga on pregnancy outcome in hypertensive disorder of pregnancy. Complement Ther Clin Pract. 2021 May;43:101366.	Wrong patient population
**19**	Perales M, Calabria I, Lopez C, Franco E, Coteron J, Barakat R. Regular Exercise Throughout Pregnancy Is Associated With a Shorter First Stage of Labor. Am J Health Promot. 2016 Jan-Feb;30(3):149-54.	Wrong outcomes
**20**	Yeo S, Steele NM, Chang MC, Leclaire SM, Ronis DL, Hayashi R. Effect of exercise on blood pressure in pregnant women with a high risk of	Full text not available

	gestational hypertensive disorders. J Reprod Med. 2000 Apr;45(4):293-8. Erratum in: J Reprod Med 2000 Jul;45(7):607. PMID: 10804484.	
**21**	Tabari, N.S. & Mirdar, Shadmehr & Khaldan, A. & Shirvani, Marjan. (2010). The Effect of Aerobic Exercise on Pregnancy Outcomes. Journal of Babol University of Medical Sciences. 12. 36-43.	languages
**22**	Newham JJ, Wittkowski A, Hurley J, Aplin JD, Westwood M. Effects of antenatal yoga on maternal anxiety and depression: a randomized controlled trial. Depress Anxiety. 2014 Aug;31(8):631-40.	Wrong outcomes
**23**	Nobles C, Marcus BH, Stanek EJ 3rd, Braun B, Whitcomb BW, Solomon CG, Manson JE, Markenson G, Chasan-Taber L. Effect of an exercise intervention on gestational diabetes mellitus: a randomized controlled trial. Obstet Gynecol. 2015 May;125(5):1195-1204.	Wrong intervention
**24**	El-Rafie MM, Khafagy GM, Gamal MG. Effect of aerobic exercise during pregnancy on antenatal depression. Int J Womens Health. 2016 Feb 24;8:53-7.	Wrong outcomes
**25**	Yekefallah L, Namdar P, Dehghankar L, Golestaneh F, Taheri S, Mohammadkhaniha F. The effect of yoga on the delivery and neonatal outcomes in nulliparous pregnant women in Iran: a clinical trial study. BMC Pregnancy Childbirth. 2021 May 3;21(1):351.	Wrong outcomes
**26**	Jayasudha A. Effect of antenatal exercises on labour outcome among primigravid mothers. Nurs J India. 2013 Jan-Feb;104(1):10-3. PMID: 23923184.	Full text not available
**27**	Coll Risco, I.; Ruiz Cabello, P.; Romero Gallardo, L.; Acosta Manzano, P.; Borges Cosic, M.; Ocon, O.; Onieva Aguayo, C.; Gascon Cortes, E.; Sanchez Gonzalez, C.; Aparicio, V. A. (2019). Influence of a Concurrent Exercise Training Intervention during Pregnancy on Maternal and Arterial and Venous Cord Serum Cytokines: The GESTAFIT Project. Journal of clinical medicine. 8. 10.3390/jcm8111862.	Wrong outcomes
**28**	Callaway LK, Colditz PB, Byrne NM, Lingwood BE, Rowlands IJ, Foxcroft K, McIntyre HD; BAMBINO Group. Prevention of gestational diabetes: feasibility issues for an exercise intervention in obese pregnant women. Diabetes Care. 2010 Jul;33(7):1457-9.	Wrong patient population
**29**	Barakat R, Ruiz JR, Stirling JR, Zakynthinaki M, Lucia A. Type of delivery is not affected by light resistance and toning exercise training during pregnancy: a randomized controlled trial. Am J Obstet Gynecol. 2009 Dec;201(6):590.e1-6.	Wrong outcomes

**30**	Kluge J, Hall D, Louw Q, Theron G, Grové D. Specific exercises to treat pregnancy-related low back pain in a South African population. Int J Gynaecol Obstet. 2011 Jun;113(3):187-91.	Wrong patient population
**31**	Dinc A, Kizilkaya Beji N, Yalcin O. Effect of pelvic floor muscle exercises in the treatment of urinary incontinence during pregnancy and the postpartum period. Int Urogynecol J Pelvic Floor Dysfunct. 2009 Oct;20(10):1223-31.	Wrong outcomes
**32**	Polley BA, Wing RR, Sims CJ. Randomized controlled trial to prevent excessive weight gain in pregnant women. Int J Obes Relat Metab Disord. 2002 Nov;26(11):1494-502.	Wrong intervention
**33**	Clark E, Isler C, Strickland D, McMillan AG, Fang X, Kuehn D, Ravisankar S, Strom C, May LE. Influence of aerobic exercise on maternal lipid levels and offspring morphometrics. Int J Obes (Lond). 2019 Mar;43(3):594-602.	Wrong outcomes
**34**	Aguilar-Cordero MJ, Sánchez-García JC, Rodriguez-Blanque R, Sánchez-López AM, Mur-Villar N. Moderate Physical Activity in an Aquatic Environment During Pregnancy (SWEP Study) and Its Influence in Preventing Postpartum Depression. J Am Psychiatr Nurses Assoc. 2019 Mar/Apr;25(2):112-121.	Wrong outcomes
**35**	Chuntharapat S, Petpichetchian W, Hatthakit U. Yoga during pregnancy: effects on maternal comfort, labor pain and birth outcomes. Complement Ther Clin Pract. 2008 May;14(2):105-15.	Wrong outcomes
**36**	Jahdi F, Sheikhan F, Haghani H, Sharifi B, Ghaseminejad A, Khodarahmian M, Rouhana N. Yoga during pregnancy: The effects on labor pain and delivery outcomes (A randomized controlled trial). Complement Ther Clin Pract. 2017 May;27:1-4.	Wrong outcomes
**37**	Bain E, Crane M, Tieu J, Han S, Crowther CA, Middleton P. Diet and exercise interventions for preventing gestational diabetes mellitus. Cochrane Database Syst Rev. 2015 Apr 12;(4):CD010443.	Wrong study design
**38**	Gustafsson MK, Stafne SN, Romundstad PR, Mørkved S, Salvesen K, Helvik AS. The effects of an exercise programme during pregnancy on health-related quality of life in pregnant women: a Norwegian randomised controlled trial. BJOG. 2016 Jun;123(7):1152-60.	Wrong outcomes
**39**	Prabha, B. S.; Vijayaraghavan, J.; Maiya, A. G.; Venkatesh, N.; Sivakumar, R. (2019). Effects of antenatal Exercise programme and education on health	Wrong outcomes

	related quality of life: A randomized control trail. Journal of Clinical and Diagnostic Research. 13. 10.7860/JCDR/2019/38086.12575.	
**40**	Bisson M, Alméras N, Dufresne SS, Robitaille J, Rhéaume C, Bujold E, Frenette J, Tremblay A, Marc I. A 12-Week Exercise Program for Pregnant Women with Obesity to Improve Physical Activity Levels: An Open Randomised Preliminary Study. PLoS One. 2015 Sep 16;10(9):e0137742.	Wrong outcomes
**41**	Carrascosa MDC, Navas A, Artigues C, Ortas S, Portells E, Soler A, Bennasar-Veny M, Leiva A; Aquanatal Trial. Effect of aerobic water exercise during pregnancy on epidural use and pain: A multi-centre, randomised, controlled trial. Midwifery. 2021 Dec;103:103105.	Wrong outcomes
**42**	Rodríguez-Blanque R, Aguilar-Cordero MJ, Marín-Jiménez AE, Menor-Rodríguez MJ, Montiel-Troya M, Sánchez-García JC. Water Exercise and Quality of Life in Pregnancy: A Randomised Clinical Trial. Int J Environ Res Public Health. 2020 Feb 17;17(4):1288.	Wrong outcomes
**43**	Kramer MS, McDonald SW. Aerobic exercise for women during pregnancy. Cochrane Database Syst Rev. 2006 Jul 19;2006(3):CD000180	Wrong study design
**44**	Salvesen KÅ, Stafne SN, Eggebø TM, Mørkved S. Does regular exercise in pregnancy influence duration of labor? A secondary analysis of a randomized controlled trial. Acta Obstet Gynecol Scand. 2014 Jan;93(1):73-9.	Wrong outcomes
**45**	Smith j. How Exercising During Pregnancy Can Boost Your Baby's Development. Sports medicine 2019	Wrong study design
**46**	Gustafsson MK, Romundstad PR, Stafne SN, Helvik AS, Stunes AK, Mørkved S, Salvesen KÅ, Thorsby PM, Mosti MP, Syversen U. The effect of an exercise program in pregnancy on vitamin D status among healthy, pregnant Norwegian women: a randomized controlled trial. BMC Pregnancy Childbirth. 2019 Feb 20;19(1):76.	Wrong outcomes
**47**	Saccone G, Berghella V, Venturella R, D'Alessandro P, Arduino B, Raffone A, Giudicepietro A, Visentin S, Vitagliano A, Martinelli P, Zullo F; Italian Preterm Birth Prevention (IPP) Working Group. Effects of exercise during pregnancy in women with short cervix: Secondary analysis from the Italian Pessary Trial in singletons. Eur J Obstet Gynecol Reprod Biol. 2018 Oct;229:132-136.	Wrong patient population
**48**	Perales M, Santos-Lozano A, Sanchis-Gomar F, Luaces M, Pareja-Galeano H, Garatachea N, Barakat R, Lucia A. Maternal Cardiac Adaptations to a	Wrong outcomes

	Physical Exercise Program during Pregnancy. Med Sci Sports Exerc. 2016 May;48(5):896-906.	
**49**	Kahyaoglu Sut H, Balkanli Kaplan P. Effect of pelvic floor muscle exercise on pelvic floor muscle activity and voiding functions during pregnancy and the postpartum period. Neurourol Urodyn. 2016 Mar;35(3):417-22.	Wrong outcomes
**50**	Barakat R, Ruiz JR, Lucia A. Exercise during pregnancy and risk of maternal anaemia: a randomised controlled trial. Br J Sports Med. 2009 Dec;43(12):954-6.	Wrong outcomes
**51**	Ramírez-Vélez R. A 12-week exercise program performed during the second trimester does not prevent gestational diabetes in healthy pregnant women. J Physiother. 2012;58(3):198.	Wrong study design
**52**	Yin YN, Li XL, Tao TJ, Luo BR, Liao SJ. Physical activity during pregnancy and the risk of gestational diabetes mellitus: a systematic review and meta-analysis of randomised controlled trials. Br J Sports Med. 2014 Feb;48(4):290-5.	Wrong study design
**53**	Oostdam N, van Poppel MN, Wouters MG, Eekhoff EM, Bekedam DJ, Kuchenbecker WK, Quartero HW, Heres MH, van Mechelen W. No effect of the FitFor2 exercise programme on blood glucose, insulin sensitivity, and birthweight in pregnant women who were overweight and at risk for gestational diabetes: results of a randomised controlled trial. BJOG. 2012 Aug;119(9):1098-107.	Wrong outcomes
**54**	Haakstad L, Bo K. Effect of supervised aerobic dance exercise in prevention of excessive weight gain in pregnancy: A single blind randomized controlled trial. Int J Gynecol Obstet. 2009;107:S198.	Wrong study design (only poster presentation available)
**55**	Hawkins M, Braun B, Marcus BH, Stanek E 3rd, Markenson G, Chasan-Taber L. The impact of an exercise intervention on C - reactive protein during pregnancy: a randomized controlled trial. BMC Pregnancy Childbirth. 2015 Jun 24;15:139.	Wrong outcomes
**56**	Vinter CA, Jensen DM, Ovesen P, Beck-Nielsen H, Jørgensen JS. The LiP (Lifestyle in Pregnancy) study: a randomized controlled trial of lifestyle intervention in 360 obese pregnant women. Diabetes Care. 2011 Dec;34(12):2502-7.	Wrong intervention
**57**	Bacchi M, Mottola MF, Perales M, Refoyo I, Barakat R. Aquatic Activities During Pregnancy Prevent Excessive Maternal Weight Gain and Preserve	Wrong outcomes

	Birth Weight: A Randomized Clinical Trial. Am J Health Promot. 2018 Mar;32(3):729-735.	
**58**	Petrov Fieril K, Glantz A, Fagevik Olsen M. The efficacy of moderate-to-vigorous resistance exercise during pregnancy: a randomized controlled trial. Acta Obstet Gynecol Scand. 2015 Jan;94(1):35-42.	Wrong outcomes
**59**	Rodríguez-Blanque R, Sánchez-García JC, Sánchez-López AM, Mur-Villar N, Fernández-Castillo R, Aguilar Cordero MJ. Influencia del ejercicio físico durante el embarazo sobre el peso del recién nacido: un ensayo clínico aleatorizado [Influence of physical exercise during pregnancy on newborn weight: a randomized clinical trial]. Nutr Hosp. 2017 Jul 28;34(4):834-840. Spanish	languages
**60**	Cavalcante SR, Cecatti JG, Pereira RI, Baciuk EP, Bernardo AL, Silveira C. Water aerobics II: maternal body composition and perinatal outcomes after a program for low risk pregnant women. Reprod Health. 2009 Jan 6;6:1.	Wrong outcomes
**61**	Daly, N.; Farren, M.; McKeating, A.; Reynolds, C. M.; Egan, B.; Turner, M. J.The effect of a medically-supervised exercise intervention for obese pregnant women on quality of life and fitness: a randomised controlled trial. Bjog 2019;126(6):e131	Wrong study design (Meeting Abstracts)
**62**	Salvesen KA, Mørkved S. Randomised controlled trial of pelvic floor muscle training during pregnancy. BMJ. 2004 Aug 14;329(7462):378-80.	Wrong outcomes
**63**	Seneviratne, S.; Cutfield, W.; Mc Cowan, L.; Ekeroma, A.; Parry, G.; Gusso, S.; Jiang, Y.; Craige, S.; Peres, G.; Rodrigues, R.; et al. Improve trial-improving maternal and progeny risk of obesity via exercise-A randomised controlled trial on effects of exercise in pregnancy in overweight and obese women and their offspring. Obesity Reviews 2014;15():124-2014	Wrong study design (abstract)
**64**	El Beltagy, N.; Saad El Deen, S.; Mohamed, R.Does physical activity and diet control reduce the risk of developing gestational diabetes mellitus in egypt? A randomized controlled trial. Journal of Perinatal Medicine 2013;41(SUPPL. 1): Walter de Gruyter GmbH and Co. KG 2013	Wrong study design (Oral presentation)
**65**	Danielli M, Gillies C, Thomas RC, Melford SE, Baker PN, Yates T, Khunti K, Tan BK. Effects of Supervised Exercise on the Development of Hypertensive Disorders of Pregnancy: A Systematic Review and Meta-Analysis. J Clin Med. 2022 Feb 1;11(3):793.	Wrong study design

**66**	Guan, Jennifer PT, DPT, MA1; Hamnett, Claire PT, DPT1; Jakucionis, Samantha PT, DPT1; Hameed, Farah MD2; Chiarello, Cynthia PT, PhD1. Can an Outpatient Exercise Program for Pregnancy-Related Pelvic Girdle Pain Improve Pain and Function Versus Education? A Feasibility Study. Journal of Women's Health Physical Therapy 45(2):p 68-75, April/June 2021.	Wrong patient population
**67**	Aguilar Cordero MJ, Rodríguez Blanque R, Sánchez García JC, Sánchez López AM, Baena García L, López Contreras G. Influencia del programa SWEP (Study Water Exercise Pregnant) en los resultados perinatales: protocolo de estudio. Nutr Hosp 2016;33:162-176	languages
**68**	Ramírez-Vélez R, Romero M, Echeverri I, Ortega JG, Mosquera M, Salazar B, Girón SL, Saldarriaga W, Aguilar de Plata AC, Mateus JC. A factorial randomized controlled trial to evaluate the effect of micronutrients supplementation and regular aerobic exercise on maternal endothelium-dependent vasodilatation and oxidative stress of the newborn. Trials. 2011 Feb 28;12:60.	Wrong outcomes
**69**	Kasawara KT, do Nascimento SL, Costa ML, Surita FG, e Silva JL. Exercise and physical activity in the prevention of pre-eclampsia: systematic review. Acta Obstet Gynecol Scand. 2012 Oct;91(10):1147-57.	Wrong study design
**70**	Sagedal LR, Sanda B, Øverby NC, Bere E, Torstveit MK, Lohne-Seiler H, Hillesund ER, Pripp AH, Henriksen T, Vistad I. The effect of prenatal lifestyle intervention on weight retention 12 months postpartum: results of the Norwegian Fit for Delivery randomised controlled trial. BJOG. 2017 Jan;124(1):111-121.	Wrong outcomes
**71**	Stafne SN, Salvesen KÅ, Romundstad PR, Torjusen IH, Mørkved S. Does regular exercise including pelvic floor muscle training prevent urinary and anal incontinence during pregnancy? A randomised controlled trial. BJOG. 2012 Sep;119(10):1270-80.	Wrong outcomes
**72**	Zarezade T. Effect of exercise on preventing gestational diabetes. IRCT201410010935N2, 2019.	Wrong study design (protocol)
**73**	Nasiri-Amiri F, Sepidarkish M, Shirvani MA, Habibipour P, Tabari NSM. The effect of exercise on the prevention of gestational diabetes in obese and overweight pregnant women: a systematic review and meta-analysis. Diabetol Metab Syndr. 2019 Aug 27;11:72.	Wrong study design
**74**	Ming WK, Ding W, Zhang CJP, Zhong L, Long Y, Li Z, Sun C, Wu Y, Chen H, Chen H, Wang Z. The effect of exercise during pregnancy on	Wrong study design

	gestational diabetes mellitus in normal-weight women: a systematic review and meta-analysis. BMC Pregnancy Childbirth. 2018 Nov 12;18(1):440.	
**75**	El-Shamy F, F, Abd El Fatah E: Effect of Antenatal Pelvic Floor Muscle Exercise on Mode of Delivery: A Randomized Controlled Trial. Integr Med Int 2017;4:187-197.	Wrong outcomes
**76**	Ghandali NY, Iravani M, Habibi A, Cheraghian B. The effectiveness of a Pilates exercise program during pregnancy on childbirth outcomes: a randomised controlled clinical trial. BMC Pregnancy Childbirth. 2021 Jul 2;21(1):480.	Wrong outcomes
**77**	Eslami, Elham & Mohammad-Alizadeh, Sakineh & farshbaf-khalili, Azizeh & Asghari Jafarabadi, Mohammad & Mirghafourvand, Mojgan. (2018). The Effect of a Lifestyle-Based Training Package on Weight Gain and Frequency of Gestational Diabetes in Obese and Overweight Pregnant Females. Iranian Red Crescent Medical Journal. In Press. 10.5812/ircmj.62576.	Wrong intervention
**78**	Niaraki, M. R.; Pakniat, H.; Alizadeh, A.; Hosseini, M. A.; Ranjkesh, F. Effect of exercise in water on the musculoskeletal pain in pregnant women: a randomized controlled trial. Journal of musculoskeletal research 2021.	Wrong outcomes
**79**	Rodríguez-Blanque R, Sánchez-García JC, Sánchez-López AM, Aguilar-Cordero MJ. Physical activity during pregnancy and its influence on delivery time: a randomized clinical trial. PeerJ. 2019 Feb 7;7:e6370.	Wrong outcomes
**80**	Field T, Diego M, Hernandez-Reif M, Medina L, Delgado J, Hernandez A. Yoga and massage therapy reduce prenatal depression and prematurity. J Bodyw Mov Ther. 2012 Apr;16(2):204-9.	Wrong outcomes
**81**	Nobles C, Marcus BH, Stanek EJ 3rd, Braun B, Whitcomb BW, Manson JE, Markenson G, Chasan-Taber L. The Effect of an Exercise Intervention on Gestational Weight Gain: The Behaviors Affecting Baby and You (B.A.B.Y.) Study: A Randomized Controlled Trial. Am J Health Promot. 2018 Mar;32(3):736-744.	Wrong intervention
**82**	Yu Y, Xie R, Shen C, Shu L. Effect of exercise during pregnancy to prevent gestational diabetes mellitus: a systematic review and meta-analysis. J Matern Fetal Neonatal Med. 2018 Jun;31(12):1632-1637.	Wrong study design
**83**	Ruchat SM, Davenport MH, Giroux I, Hillier M, Batada A, Sopper MM, McManus R, Hammond JA, Mottola MF. Effect of exercise intensity and duration on capillary glucose responses in pregnant women at low and high	Wrong outcomes

	risk for gestational diabetes. Diabetes Metab Res Rev. 2012 Nov;28(8):669-78.	
**84**	Ramirez-Velez, R. Effects of endurance training on expression endothelial nitric oxide synthase and nitric oxide production in human placenta. Circulation 2012;125(19):e916. Lippincott Williams and Wilkins 2012	Wrong study design (poster presentation)
**85**	Cruz, C.; Riesco, M. L.; Zanetti, M. Supervised pelvic floor muscle training to treat urinary incontinence during pregnancy: a randomized controlled trial. Neurourology and Urodynamics 2014;33(6):867-868. 2014	Wrong outcomes
**86**	Barakat R, Pelaez M, Montejo R, Luaces M, Zakynthinaki M. Exercise during pregnancy improves maternal health perception: a randomized controlled trial. Am J Obstet Gynecol. 2011 May;204(5):402.e1-7.	Wrong outcomes
**87**	Zheng J, Wang H, Ren M. Influence of exercise intervention on gestational diabetes mellitus: a systematic review and meta-analysis. J Endocrinol Invest. 2017 Oct;40(10):1027-1033.	Wrong study design
**88**	Beddoe AE, Paul Yang CP, Kennedy HP, Weiss SJ, Lee KA. The effects of mindfulness-based yoga during pregnancy on maternal psychological and physical distress. J Obstet Gynecol Neonatal Nurs. 2009 May-Jun;38(3):310-9.	Wrong study design
**89**	Embaby H, Elsayed E, Fawzy M. Insulin Sensitivity and Plasma Glucose Response to Aerobic Exercise in Pregnant Women at Risk for Gestational Diabetes Mellitus. Ethiop J Health Sci. 2016 Sep;26(5):409-414.	Wrong outcomes
**90**	Bolanthakodi C, Raghunandan C, Saili A, Mondal S, Saxena P. Prenatal Yoga: Effects on Alleviation of Labor Pain and Birth Outcomes. J Altern Complement Med. 2018 Dec;24(12):1181-1188.	Wrong outcomes
**91**	McDonald SM, Strom C, Remchak MM, Chaves A, Broskey NT, Isler C, Haven K, Newton E, DeVente J, Acosta-Manzano P, Aparicio VA, May LE. The effects of aerobic exercise on markers of maternal metabolism during pregnancy. Birth Defects Res. 2021 Feb 1;113(3):227-237.	Wrong outcomes
**92**	Van Poppel, M.; Oostdam, N.; Wouters, M.; Eekhoff, M.; Van Mechelen, W. FitFor2: Effects of an exercise training program on the incidence of gestational diabetes. Journal of Science and Medicine in Sport 2012;15(SUPPL.1):S342-S343. Elsevier Ltd 2012	Wrong study design
**93**	Oostdam N, Bosmans J, Wouters MG, Eekhoff EM, van Mechelen W, van Poppel MN. Cost-effectiveness of an exercise program during pregnancy to	Wrong outcomes

	prevent gestational diabetes: results of an economic evaluation alongside a randomised controlled trial. BMC Pregnancy Childbirth. 2012 Jul 4;12:64.	
**94**	Bahadoran P, Pouya F, Zolaktaf V, Taebi M. The effect of stretching exercise and walking on changes of blood pressure in nulliparous women. Iran J Nurs Midwifery Res. 2015 Mar-Apr;20(2):205-10.	Wrong outcomes
**95**	Agur W, Steggles P, Waterfield M, Freeman R. Does antenatal pelvic floor muscle training affect the outcome of labour? A randomised controlled trial. Int Urogynecol J Pelvic Floor Dysfunct. 2008 Jan;19(1):85-8.	Wrong outcomes
**96**	Taniguchi C, Sato C. Home-based walking during pregnancy affects mood and birth outcomes among sedentary women: A randomized controlled trial. Int J Nurs Pract. 2016 Oct;22(5):420-426.	Wrong intervention
**97**	Tomić V, Sporiš G, Tomić J, Milanović Z, Zigmundovac-Klaić D, Pantelić S. The effect of maternal exercise during pregnancy on abnormal fetal growth. Croat Med J. 2013 Aug;54(4):362-8.	Duplet
**98**	Abedi Amiri, M., Avandi, S. M., Esmaeilzadeh, S. Effect of six-week pranayama training on the serum levels of cortisol and blood pressure in pregnant women in the third trimester. *The Iranian Journal of Obstetrics, Gynecology and Infertility*, 2018; 21(6): 55-63.	languages
**99**	Wolfe LA, Walker RM, Bonen A, McGrath MJ. Effects of pregnancy and chronic exercise on respiratory responses to graded exercise. J Appl Physiol (1985). 1994 May;76(5):1928-36.	Wrong outcomes
**100**	Aklar Corekci, A.; Avci, O.; Takinaci, Z. D. Is pregnancy yoga effective in pregnant women in terms of static balance? Fizyoterapi Rehabilitasyon 2017;28(2):S59 Netherlands Turkish Physiotherapy Association 2017	Wrong outcomes
**101**	Ramírez-Vélez R, Aguilar AC, Mosquera M, Garcia RG, Reyes LM, López-Jaramillo P. Clinical trial to assess the effect of physical exercise on endothelial function and insulin resistance in pregnant women. Trials. 2009 Nov 17;10:104.	Wrong outcomes
**102**	Rakhshani A, Maharana S, Raghuram N, Nagendra HR, Venkatram P. Effects of integrated yoga on quality of life and interpersonal relationship of pregnant women. Qual Life Res. 2010 Dec;19(10):1447-55.	Wrong outcomes
**103**	Ferreira CLM, Guerra CML, Silva AITJ, do Rosário HRV, Pereira MBFLO. Exercise in Pregnancy: The Impact of an Intervention Program in the Duration of Labor and Mode of Delivery. Rev Bras Ginecol Obstet. 2019 Feb;41(2):68-75. English.	Wrong patient population

**104**	Makaruk B, Iciek R, Zalewski A, Galczak-Kondraciuk A, Grantham W. The effects of a physical exercise program on fetal well-being and intrauterine safety. Ginekol Pol. 2021;92(2):126-131.	Wrong outcomes
**105**	Nascimento SL, Surita FG, Parpinelli MÂ, Siani S, Pinto e Silva JL. The effect of an antenatal physical exercise programme on maternal/perinatal outcomes and quality of life in overweight and obese pregnant women: a randomised clinical trial. BJOG. 2011 Nov;118(12):1455-63.	Wrong patient population
**106**	Marquez-Sterling S, Perry AC, Kaplan TA, Halberstein RA, Signorile JF. Physical and psychological changes with vigorous exercise in sedentary primigravidae. Med Sci Sports Exerc. 2000 Jan;32(1):58-62.	Wrong outcomes
**107**	Barakat R, Stirling JR, Lucia A. Does exercise training during pregnancy affect gestational age? A randomised controlled trial. Br J Sports Med. 2008 Aug;42(8):674-8.	Wrong outcomes
**108**	Bø K, Haakstad LA. Is pelvic floor muscle training effective when taught in a general fitness class in pregnancy? A randomised controlled trial. Physiotherapy. 2011 Sep;97(3):190-5.	Wrong outcomes
**109**	Garnæs KK, Helvik AS, Stafne SN, Mørkved S, Salvesen K, Salvesen Ø, Moholdt T. Effects of supervised exercise training during pregnancy on psychological well-being among overweight and obese women: secondary analyses of the ETIP-trial, a randomised controlled trial. BMJ Open. 2019 Nov 21;9(11):e028252	Wrong outcomes
**110**	Deng Y, Hou Y, Wu L, Liu Y, Ma L, Yao A. Effects of Diet and Exercise Interventions to Prevent Gestational Diabetes Mellitus in Pregnant Women With High-Risk Factors in China: A Randomized Controlled Study. Clin Nurs Res. 2022 Jun;31(5):836-847.	Wrong intervention
**111**	Wang, C.; Wei, Y.; Zhang, X.; Zhang, Y.; Xu, Q.; Su, S.; Zhang, L.; Liu, C.; Feng, Y.; Shou, C.; Guelfi, K. J.; Newnham, J. P.; Yang, H. Effect of Regular Exercise Commenced in Early Pregnancy on the Incidence of Gestational Diabetes Mellitus in Overweight and Obese Pregnant Women: A Randomized Controlled Trial. Diabetes Care Oct 2016;39(10):e163-4. 2016 Oct	Wrong study design (abstract)
**112**	Ji ES, Han HR. The effects of Qi exercise on maternal/fetal interaction and maternal well-being during pregnancy. J Obstet Gynecol Neonatal Nurs. 2010 May-Jun;39(3):310-8.	Wrong outcomes

**113**	Holden SC, Manor B, Zhou J, Zera C, Davis RB, Yeh GY. Prenatal Yoga for Back Pain, Balance, and Maternal Wellness: A Randomized, Controlled Pilot Study. Glob Adv Health Med. 2019 Aug 26;8:2164956119870984.	Wrong outcomes
**114**	Eggen MH, Stuge B, Mowinckel P, Jensen KS, Hagen KB. Can supervised group exercises including ergonomic advice reduce the prevalence and severity of low back pain and pelvic girdle pain in pregnancy? A randomized controlled trial. Phys Ther. 2012 Jun;92(6):781-90.	Wrong outcomes
**115**	Fournier, D, Feeney, G, Mathieu, M. Outcomes of Exercise training following the use of a birthing Ball during pregnancy and Delivery. J Strength Cond Res 31: 1942–1948, 2017.	Wrong outcomes
**116**	Dias NT, Ferreira LR, Fernandes MG, Resende APM, Pereira-Baldon VS. A Pilates exercise program with pelvic floor muscle contraction: Is it effective for pregnant women? A randomized controlled trial. Neurourol Urodyn. 2018 Jan;37(1):379-384.	Wrong outcomes
**117**	Haakstad LA, Edvardsen E, Bø K. Effect of regular exercise on blood pressure in normotensive pregnant women. A randomized controlled trial. Hypertens Pregnancy. 2016 May;35(2):170-80.	Wrong outcomes
**118**	Moholdt T, Silva CP, Lydersen S, Hawley JA. Isolated and combined effects of high-intensity interval training and time-restricted eating on glycaemic control in reproductive-aged women with overweight or obesity: study protocol for a four-armed randomised controlled trial. BMJ Open. 2021 Feb 5;11(2):e040020.	Wrong study design (protocol)
**119**	Babbar S, Hill JB, Williams KB, Pinon M, Chauhan SP, Maulik D. Acute feTal behavioral Response to prenatal Yoga: a single, blinded, randomized controlled trial (TRY yoga). Am J Obstet Gynecol. 2016 Mar;214(3):399.e1-8.	Wrong outcomes
**120**	Skow RJ, Labrecque L, Rosenberger JA, Brassard P, Steinback CD, Davenport MH. Prenatal exercise and cardiovascular health (PEACH) study: impact of acute and chronic exercise on cerebrovascular hemodynamics and dynamic cerebral autoregulation. J Appl Physiol (1985). 2022 Jan 1;132(1):247-260.	Wrong outcomes
**121**	Yeo S. A randomized comparative trial of the efficacy and safety of exercise during pregnancy: design and methods. Contemp Clin Trials. 2006 Dec;27(6):531-40.	Wrong outcomes

**122**	Han S, Middleton P, Crowther CA. Exercise for pregnant women for preventing gestational diabetes mellitus. Cochrane Database of Systematic Reviews 2012, Issue 7. Art. No.: CD009021.	Wrong study design
**123**	Sklempe Kokic I, Ivanisevic M, Uremovic M, Kokic T, Pisot R, Simunic B. Effect of therapeutic exercises on pregnancy-related low back pain and pelvic girdle pain: Secondary analysis of a randomized controlled trial. J Rehabil Med. 2017 Mar 6;49(3):251-257.	Wrong outcomes
**124**	Pinzón DC, Zamora K, Martínez JH, Floréz-López ME, de Plata AC, Mosquera M, Ramírez-Vélez R. Type of delivery and gestational age is not affected by pregnant Latin-American women engaging in vigorous exercise: a secondary analysis of data from a controlled randomized trial. Rev Salud Publica (Bogota). 2012 Oct;14(5):731-43. PMID: 24652353.	Wrong outcomes
**125**	Guelfi, K. J.; Ong, M. J.; Fournier, P. A.; Wallman, K. E.; Grove, J. R.; Doherty, D. A.; Newnham, J. P.<br>Does supervised home-based exercise during pregnancy reduce the recurrence of gestational diabetes? A randomised controlled trial. Journal of Paediatrics and Child Health 2015;51(SUPPL. 1):35<br>Blackwell Publishing 2015	Full text not available
**126**	Ko PC, Liang CC, Chang SD, Lee JT, Chao AS, Cheng PJ. A randomized controlled trial of antenatal pelvic floor exercises to prevent and treat urinary incontinence. Int Urogynecol J. 2011 Jan;22(1):17-22.	Wrong outcomes
**127**	Santos IA, Stein R, Fuchs SC, Duncan BB, Ribeiro JP, Kroeff LR, Carballo MT, Schmidt MI. Aerobic exercise and submaximal functional capacity in overweight pregnant women: a randomized trial. Obstet Gynecol. 2005 Aug;106(2):243-9.	Wrong outcomes
**128**	Stuge B. Group training reduces the risk of pregnancy-related lumbopelvic pain. Aust J Physiother. 2007;53(3):202.	Wrong outcomes
**129**	Van Poppel, M. N.; Oostdam, N.; Wouters, M. G.; Eekhoff, M. M.; Van Mechelen, W. A training program for women at risk for gestational diabetes. Diabetes 2011;60(SUPPL. 1):A636 American Diabetes Association Inc. 2011	Wrong study design (abstract)
**130**	Deshpande CS, Rakhshani A, Nagarathna R, Ganpat TS, Kurpad A, Maskar R, Nagendra HR, Sudheer DC, Abbas R, Raghuram N, Anura K, Rita M, Ramarao N. Yoga for high-risk pregnancy: a randomized controlled trial. Ann Med Health Sci Res. 2013 Jul;3(3):341-4.	Wrong patient population

**131**	Garshasbi A, Faghih Zadeh S. The effect of exercise on the intensity of low back pain in pregnant women. Int J Gynaecol Obstet. 2005 Mar;88(3):271-5.	Wrong outcomes
**132**	Sangsawang B, Sangsawang N. Is a 6-week supervised pelvic floor muscle exercise program effective in preventing stress urinary incontinence in late pregnancy in primigravid women?: a randomized controlled trial. Eur J Obstet Gynecol Reprod Biol. 2016 Feb;197:103-10.	Wrong outcomes
**133**	Satyapriya M, Nagarathna R, Padmalatha V, Nagendra HR. Effect of integrated yoga on anxiety, depression & well being in normal pregnancy. Complement Ther Clin Pract. 2013 Nov;19(4):230-6.	Wrong outcomes
**134**	Rodriguez-Blanque R, Sánchez-García JC, Sánchez-López AM, Mur-Villar N, Aguilar-Cordero MJ. The influence of physical activity in water on sleep quality in pregnant women: A randomised trial. Women Birth. 2018 Feb;31(1):e51-e58.	Wrong outcomes
**135**	Fahey MC, Wayne Talcott G, Cox Bauer CM, Bursac Z, Gladney L, Hare ME, Harvey J, Little M, McCullough D, Hryshko-Mullen AS, Klesges RC, Kocak M, Waters TM, Krukowski RA. Moms fit 2 fight: Rationale, design, and analysis plan of a behavioral weight management intervention for pregnant and postpartum women in the U.S. military. Contemp Clin Trials. 2018 Nov;74:46-54.	Wrong intervention
**136**	Skow RJ, Steinback CD, Davenport MH. Prenatal Exercise and Cardiovascular Health (PEACH) Study: Impact on the Vascular System. Med Sci Sports Exerc. 2021 Dec 1;53(12):2605-2617.	Wrong outcomes
**137**	Bacchi M, Mottola MF, Perales M, Refoyo I, Barakat R. Aquatic Activities During Pregnancy Prevent Excessive Maternal Weight Gain and Preserve Birth Weight: A Randomized Clinical Trial. Am J Health Promot. 2018 Mar;32(3):729-735.	Wrong outcomes
**138**	Bhartia, N.; Jain, S.; Shankar, N.; Rajaram, S.; Gupta, M. Effects of antenatal yoga on maternal stress and clinical outcomes in north indian women: A randomised controlled trial. Journal, Indian Academy of Clinical Medicine 2019;20(1):10-14. India Indian Academy of Clinical Medicine 2019	Wrong outcomes
**139**	Toosi m. Investigation of the effect of a cycle of exercises on pregnancy outcomes in Primigravida Women. IRCT201403167497N3. 2015.	Wrong study design (protocol)
**140**	Coll CVN, Domingues MR, Stein A, da Silva BGC, Bassani DG, Hartwig FP, da Silva ICM, da Silveira MF, da Silva SG, Bertoldi AD. Efficacy of	Wrong outcomes

	Regular Exercise During Pregnancy on the Prevention of Postpartum Depression: The PAMELA Randomized Clinical Trial. JAMA Netw Open. 2019 Jan 4;2(1):e186861.	
**141**	Mohammadi F, Malakooti J, Babapoor J, Mohammad-Alizadeh-Charandabi S. The effect of a home-based exercise intervention on postnatal depression and fatigue: A randomized controlled trial. Int J Nurs Pract. 2015 Oct;21(5):478-85.	Wrong outcomes
**142**	Zhang R, Xiao Y, Wei W, Wu B. Effect of birth ball abdominal core training on pregnancy fatigue, waist pain and delivery outcomes. Int J Gynaecol Obstet. 2022 Sep;158(3):613-618.	Wrong outcomes
**143**	Hallal c. p. Effects of Exercise During Pregnancy on Maternal and Child Health: a Randomized Clinical Trial. NCT0214896. 2014	Wrong study design (protocol)
**144**	Sanda B, Vistad I, Sagedal LR, Haakstad LAH, Lohne-Seiler H, Torstveit MK. Effect of a prenatal lifestyle intervention on physical activity level in late pregnancy and the first year postpartum. PLoS One. 2017 Nov 27;12(11):e0188102.	Full text not available
**145**	Navas A, Carrascosa MDC, Artigues C, Ortas S, Portells E, Soler A, Yañez AM, Bennasar-Veny M, Leiva A. Effectiveness of Moderate-Intensity Aerobic Water Exercise during Pregnancy on Quality of Life and Postpartum Depression: A Multi-Center, Randomized Controlled Trial. J Clin Med. 2021 May 30;10(11):2432.	Wrong outcomes
**146**	Silva-Jose C, Sánchez-Polán M, Díaz-Blanco Á, Pérez-Medina T, Carrero Martínez V, Alzola I, Barakat R, Refoyo I, Mottola MF. Influence of a Virtual Exercise Program throughout Pregnancy during the COVID-19 Pandemic on Perineal Tears and Episiotomy Rates: A Randomized Clinical Trial. J Clin Med. 2021 Nov 11;10(22):5250.	Wrong outcomes
**147**	Jayashree R, Malini A, Rakhshani A, Nagendra H, Gunasheela S, Nagarathna R. Effect of the integrated approach of yoga therapy on platelet count and uric acid in pregnancy: A multicenter stratified randomized single-blind study. Int J Yoga. 2013 Jan;6(1):39-46.	Wrong patient population
**148**	Hui, A.; Ludwig, S.; Gardiner, P.; Sevenhuysen, G.; Dean, H.; Sellers, E.; Bruce, E.; Morris, M.; Shen, G. Effect of a community-based lifestyle intervention on physical activity and diet in pregnant women. Canadian Journal of Diabetes 2009;33(3):263. Canadian Diabetes Association 2009	Wrong study design
**149**	Ong MJ, Guelfi KJ, Hunter T, Wallman KE, Fournier PA, Newnham JP. Supervised home-based exercise may attenuate the decline of glucose	Wrong outcomes

	tolerance in obese pregnant women. Diabetes Metab. 2009 Nov;35(5):418-21.	
**150**	Dias LA, Driusso P, Aita DL, Quintana SM, Bø K, Ferreira CH. Effect of pelvic floor muscle training on labour and newborn outcomes: a randomized controlled trial. Rev Bras Fisioter. 2011 Nov-Dec;15(6):487-93.	Wrong Intervention
**151**	Haakstad LA, Bø K. Effect of a regular exercise programme on pelvic girdle and low back pain in previously inactive pregnant women: A randomized controlled trial. J Rehabil Med. 2015 Mar;47(3):229-34.	Wrong outcomes
**152**	Motaghi Dastenaei B. Pilates effect on pregnancy outcomes. Irct20170124032161N. 2019	Wrong study design (study protocol)
**153**	Yeo S, Davidge ST. Possible beneficial effect of exercise, by reducing oxidative stress, on the incidence of preeclampsia. J Womens Health Gend Based Med. 2001 Dec;10(10):983-9.	Wrong study design
**154**	Robledo-Colonia AF, Sandoval-Restrepo N, Mosquera-Valderrama YF, Escobar-Hurtado C, Ramírez-Vélez R. Aerobic exercise training during pregnancy reduces depressive symptoms in nulliparous women: a randomised trial. J Physiother. 2012;58(1):9-15.	Wrong outcome
**155**	Pelaez M, Gonzalez-Cerron S, Montejo R, Barakat R. Pelvic floor muscle training included in a pregnancy exercise program is effective in primary prevention of urinary incontinence: a randomized controlled trial. Neurourol Urodyn. 2014 Jan;33(1):67-71.	Wrong outcome
**156**	Vargas-Terrones M, Barakat R, Santacruz B, Fernandez-Buhigas I, Mottola MF. Physical exercise programme during pregnancy decreases perinatal depression risk: a randomised controlled trial. Br J Sports Med. 2019 Mar;53(6):348-353.	Wrong outcome
**157**	Mørkved S, Salvesen KA, Schei B, Lydersen S, Bø K. Does group training during pregnancy prevent lumbopelvic pain? A randomized clinical trial. Acta Obstet Gynecol Scand. 2007;86(3):276-82.	Wrong outcome
**158**	Hall DC, Kaufmann DA. Effects of aerobic and strength conditioning on pregnancy outcomes. Am J Obstet Gynecol. 1987 Nov;157(5):1199-203. doi: 10.1016/s0002-9378(87)80294-6. PMID: 3688075.	Full text not available
**159**	Stafne SN, Salvesen KÅ, Romundstad PR, Eggebø TM, Carlsen SM, Mørkved S. Regular exercise during pregnancy to prevent gestational	Wrong outcome

	diabetes: a randomized controlled trial. Obstet Gynecol. 2012 Jan;119(1):29-36.	
**160**	Perales M, Valenzuela PL, Barakat R, Cordero Y, Peláez M, López C, Ruilope LM, Santos-Lozano A, Lucia A. Gestational Exercise and Maternal and Child Health: Effects until Delivery and at Post-Natal Follow-up. J Clin Med. 2020 Jan 31;9(2):379.	Data is presented earlier in Ruiz et al.
**161**	Sanda B, Vistad I, Sagedal LR, Haakstad LAH, Lohne-Seiler H, Torstveit MK. What is the effect of physical activity on duration and mode of delivery? Secondary analysis from the Norwegian Fit for Delivery trial. Acta Obstet Gynecol Scand. 2018 Jul;97(7):861-871.	Data is also presented in Sagedal et al. 2017.
**162**	Garnæs KK, Mørkved S, Salvesen Ø, Moholdt T. Exercise Training and Weight Gain in Obese Pregnant Women: A Randomized Controlled Trial (ETIP Trial). PLoS Med. 2016 Jul 26;13(7):e1002079.	Wrong patient population
**163**	Raper MJ, McDonald S, Johnston C, Isler C, Newton E, Kuehn D, Collier D, Broskey NT, Muldrow A, May LE. The influence of exercise during pregnancy on racial/ethnic health disparities and birth outcomes. BMC Pregnancy Childbirth. 2021 Mar 26;21(1):258.	Data is also presented in McDonald et al. 2021.
**164**	Haas Iii, William C.; Barakat, Ruben; Pelaez, Mireia; Cordero, Yaiza; Perales, Maria; Lopez, Carmina; Coteron, Javier; Mottola, Michelle F. Exercise during pregnancy protects against hypertension and macrosomia: randomized clinical trial. Integrative Medicine Alert 2016;19(4):37-39 2016	Wrong patient population (Data is also presented in Barakat et al. 2016.)
**165**	McMillan AG, May LE, Gaines GG, Isler C, Kuehn D. Effects of Aerobic Exercise during Pregnancy on 1-Month Infant Neuromotor Skills. Med Sci Sports Exerc. 2019 Aug;51(8):1671-1676.	Wrong outcome
**166**	Nyrnes SA, Garnæs KK, Salvesen Ø, Timilsina AS, Moholdt T, Ingul CB. Cardiac function in newborns of obese women and the effect of exercise during pregnancy. A randomized controlled trial. PLoS One. 2018 Jun 1;13(6):e0197334.	Wrong Patient population (Same study as Garnæs et al. 2016.)
**167**	Barakat R, Lucia A, Ruiz JR. Resistance exercise training during pregnancy and newborn's birth size: a randomised controlled trial. Int J Obes (Lond). 2009 Sep;33(9):1048-57.	Wrong patient population
